# Efficiency Enhancement of Hybrid Perovskite Solar Cells with MEH-PPV Hole-Transporting Layers

**DOI:** 10.1038/srep34319

**Published:** 2016-10-04

**Authors:** Hsin-Wei Chen, Tzu-Yen Huang, Ting-Hsiang Chang, Yoshitaka Sanehira, Chung-Wei Kung, Chih-Wei Chu, Masashi Ikegami, Tsutomu Miyasaka, Kuo-Chuan Ho

**Affiliations:** 1 Department of Chemical Engineering, National Taiwan University, Taipei 10617, Taiwan; 2 Research Center for Applied Sciences, Academia Sinica, Taipei 11529, Taiwan; 3 Graduate School of Engineering, Toin University of Yokohama, Yokohama 1614, Japan

## Abstract

In this study, hybrid perovskite solar cells are fabricated using poly[2-methoxy-5-(2-ethylhexyloxy)-1,4-phenylenevinylene] (MEH-PPV) and poly(3-hexylthiophene-2,5-diyl) (P3HT) as dopant-free hole-transporting materials (HTMs), and two solution processes (one- and two-step methods, respectively) for preparing methylammonium lead iodide perovskite. By optimizing the concentrations and solvents of MEH-PPV solutions, a power conversion efficiency of 9.65% with hysteresis-less performance is achieved, while the device with 2,2′,7,7′-tetrakis(N,N-di-p-methoxyphenylamine)-9,9′spirobifluorene (Spiro-OMeTAD) doped with lithium salts and tert-butylpyridine (TBP) exhibits an efficiency of 13.38%. This result shows that non-doped MEH-PPV is a suitable, low-cost HTM for efficient polymer-based perovskite solar cells. The effect of different morphologies of methylammonium lead iodide perovskite on conversion efficiency is also investigated by incident photon-to-electron conversion efficiency (IPCE) curves and electrochemical impedance spectroscopy (EIS).

In recent years, hybrid organolead halide perovskite solar cells have attracted significant attention, attributed to their inherent facile fabrication, potential cost-effectiveness, and high power conversion efficiency (PCE) reaching ~20%^1^,^2^,^3^,^4^,^5^. In 2009, Miyasaka *et al*. first reported a solar cell incorporating a perovskite absorber, and this perovskite-sensitized solar cell containing a liquid electrolyte exhibits a conversion efficiency of 3.8%^6^. In 2011, Park *et al*. reported a further increase in the conversion efficiency to 6.5%^7^. However, because the liquid electrolyte is corrosive, the perovskite material dissolves within a few minutes of device operation; this prompts a shift towards solid-state devices consisting of hole conductors such as 2,2′,7,7′-tetrakis(N, N-di-p-methoxyphenylamine)-9,9′spirobifluorene (Spiro-OMeTAD)^1^, poly(3-hexylthiophene-2,5-diyl) (P3HT)^8^,^9^, poly-(2,1,3-benzothiadiazole-4,7-diyl(4,4-bis(2-ethylhexyl)-4H-cyclopenta)2,1-b:3,4-b′)dithiophene-2,6-diyl)) (PCPDTBT)^10^, and poly-triarylamine (PTAA)^4^,^10^, and other hole-transporting materials (HTMs)^11^,^12^,^13^,^14^,^15^,^16^. Spiro-OMeTAD is the most commonly used HTM, which exhibits efficient charge transport, low recombination rates, and good pore filling of the TiO_2_ layer; these characteristics enhance device performance with respect to polymer-based HTMs^17^,^18^. As a small-molecule HTM, pristine Spiro-OMeTAD exhibits low mobility (approximately 10^−6^ to 10^−5^ cm^2^ V^−1^s^−1^)^19^,^20^. The hole conductivity of a Spiro-OMeTAD film has been reported to be improved significantly by an order of magnitude through doping with a cobalt electrolyte and lithium salts under air oxidation^21^,^22^,^23^,^24^,^25^. Nevertheless, precise control of these poorly defined oxidative processes is challenging, due to the lack of physical understanding and device reproducibility. Additionally, oxidation-induced efficiency enhancements decline after the devices are encapsulated/stored under inert atmosphere, thus preventing from the real applications^26^. Furthermore, some dopants are hydrophilic, which compromises solar cell stability considering that the perovskite active layers are susceptible to moisture-induced degradation^27^. Consequently, developing dopant-free, hydrophobic/O_2_-stable HTMs is critical for advancing perovskite solar cell technologies^28^. However, for a conducting polymer, van der Waals interactions between polymer chains (as well as those between polymer and solvent molecules in solution-processed films) are generally thought to determine the manner in which the chains are packed in the film state^29^. In fact, the film morphology developed during processing significantly affects inter-chain hopping and device performance. Besides, the size of the polymeric chain can reduce the pore filling of the mesoporous layer, thereby limiting conversion efficiency^30^. Some studies have demonstrated that the electronic properties of conjugated polymers possibly depend on film processing conditions, such as solvent, solution concentration, additives, and methods or conditions of film preparation^31^.

In this study, poly[2-methoxy-5-(2-ethylhexyloxy)-1,4-phenylenevinylene] (MEH-PPV) is demonstrated to be a suitable HTM for efficient perovskite-based solar cells. A fluorine-doped tin oxide (FTO) substrate/TiO_2_/CH_3_NH_3_PbI_3_/MEH-PPV/Au architecture is proposed with the aim of optimizing the performance of MEH-PPV-based perovskite solar cells by controlling the solvents and solution concentration. Although Masi *et al*. have reported an MEH-PPV with perovskite for fabricating an optoelectronic device with polymer or perovskite nanocomposites^32^, which exhibits an inferior efficiency of 3%, the structure of the devices in their study is significantly different from those fabricated herein. Their device structure is similar to that of the bulk hetero-junction solar cells by exploiting polymers as the template agent and forming perovskite inside the polymer matrix as the nanocomposite film, which is obtained from a MEH-PPV, methylammonium iodide, and lead iodide blend solution in a binary mixture of solvents tetrahydrofurane (THF) and N,N-dimethylformamide (DMF)^32^. For the first time, the combination of dopant-free MEH-PPV and CH_3_NH_3_PbI_3_ is used in the present study, attaining a final device efficiency of 9.65%; to the best of our knowledge, this efficiency is the highest reported value for a solar cell using MEH-PPV as an HTM.

## Results

**Figure 1:**
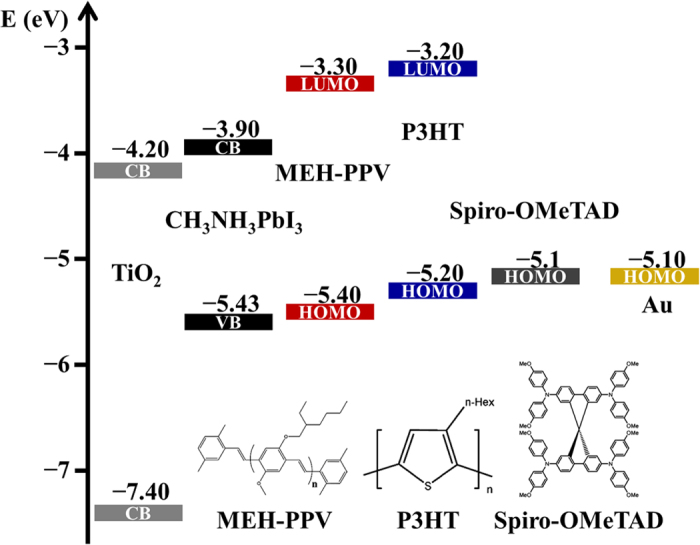
Energy band diagram and molecular structures of MEH-PPV, P3HT, and Spiro-OMeTAD.

**Figure 2:**
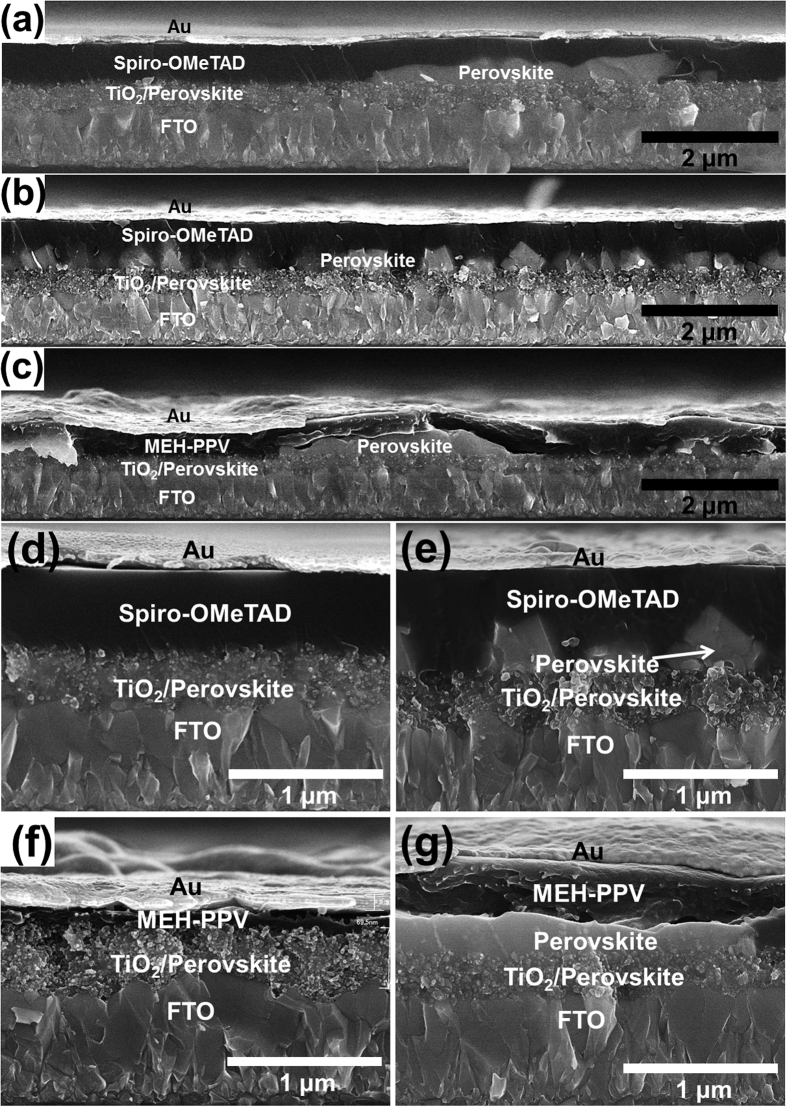
Cross-sectional SEM images of the layers observed in perovskite solar cells fabricated by the (**a,d**) one-step method of preparing CH_3_NH_3_PbI_3−x_Cl_x_ and (**b,e**) two-step method of preparing CH_3_NH_3_PbI_3_ (tetragonal structure) with Spiro-OMeTAD (15 mg mL^−1^), (**c,g**) MEH-PPV (10 mg mL^−1^), and (**f**) MEH-PPV (5 mg mL^−1^).

Perovskite solar cells with different HTMs were fabricated under ambient air conditions; details are described in methods part. Figure 1 shows the energy band diagram and chemical structures of MEH-PPV, P3HT, and Spiro-OMeTAD. For devices of the type FTO/TiO_2_/perovskite/HTMs/Au, the open-circuit voltage (*V*_*OC*_) under illumination of 1 sun is determined by the difference between the TiO_2_ quasi-Fermi energy level of ~3.2 eV and the work function of the Au electrode or the HOMO level of the HTMs. In our system, the Au electrode work function of −5.1 eV forms a nearly Ohmic contact with the HOMO level of HTMs −5.4 to −5.1 eV. The HOMO levels of polymer-based MEH-PPV and P3HT were slightly lower than that of Spiro-OMeTAD, indicating that *V*_*OC*_ is possibly higher in these two HTMs. Figure 2 shows the cross-sectional scanning electron microscopy (SEM) images of the layers observed for the perovskite solar cells fabricated by the one-step method (CH_3_NH_3_PbI_3−x_Cl_x_) and two-step method (CH_3_NH_3_PbI_3_) (tetragonal structure) using Spiro-OMeTAD and MEH-PPV as the HTMs. The polymer-based MEH-PPV layer (Fig. 2c,f,g) exhibited morphologies significantly different from those of the small-molecular-based Spiro-OMeTAD layer (Fig. 2a,b,d,e). As compared to the MEH-PPV layer, the Spiro-OMeTAD layer was significantly more smooth and compact, attributed to the small molecular property. Besides, both Spiro-OMeTAD (Fig. 2a) and MEH-PPV (10 mg mL^−1^) layers (Fig. 2c) with thicknesses of approximately 350 nm completely covered the perovskite/TiO_2_ layers; however, a lower MEH-PPV concentration (5 mg mL^−1^) resulted in a thinner layer (Fig. 2f). In a typical one-step method, a mesoporous TiO_2_ nanocrystal layer was deposited above a TiO_2_ compact layer pre-deposited on a ~300 nm thick FTO, followed by spin-coating with an organo-lead halide perovskite precursor solution (CH_3_NH_3_I and PbCl_2_ in DMF); the spin-coated perovskite/TiO_2_ layer was dried at 100 °C (Fig. 2a). However, in the two-step method, lead iodide (PbI_2_) was first introduced into the TiO_2_ nanopores by spin-coating a 1 M solution of PbI_2_ in DMF, followed by drying at 70 °C. Subsequently, the TiO_2_/PbI_2_ composite film was dipped into a solution of CH_3_NH_3_I in 2-propanol (10 mg mL^−1^), and the color immediately changed from yellow to dark brown, indicating the formation of CH_3_NH_3_PbI_3_. From Fig. 2b, e, the two-step method resulted in the formation of a capping layer with a tetragonal structure significantly different from that produced by the one-step method. The perovskite tetragonal crystals were significantly greater than the TiO_2_ nanoparticles (~400 nm); hence, perovskite is not a thin conformal layer covering the TiO_2_ particles sandwiched by Spiro-OMeTAD. As can be observed in Fig. 2e, the TiO_2_ pores were filled with PbI_2_, and a capping layer of PbI_2_ was also formed during the first coating step. During the second step in which the TiO_2_/PbI_2_ composed film was dipped into a CH_3_NH_3_I solution, PbI_2_ was completely converted to perovskite, as confirmed by X-ray diffraction (XRD) (Figure S1 in the Supplementary Information). Hence, perovskite forms not only inside the TiO_2_ pores but also on the surface of the TiO_2_ layer. Because the TiO_2_ film has a small pore size, the growth of perovskite is limited inside the mesopores; however, larger crystal growth is possible on the top of the TiO_2_ film.

**Figure 3:**
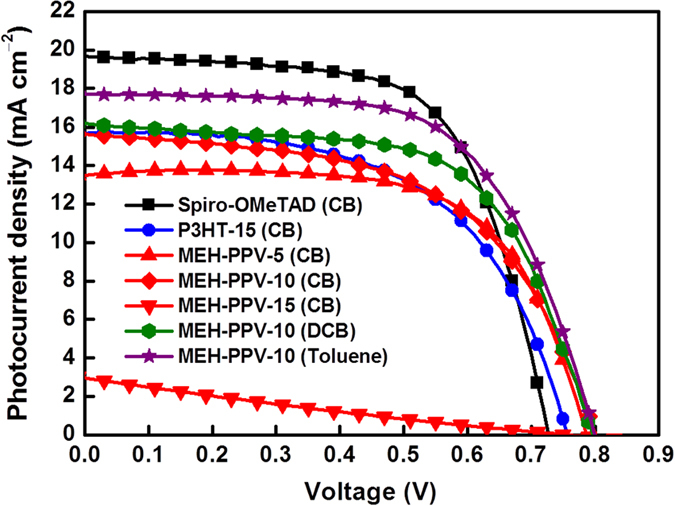
Current density–voltage (*J–V*) characteristic curves of perovskite solar cells with Spiro-OMeTAD, P3HT, and MEH-PPV HTMs dissolved in various solvents as well as concentrations.

**Figure 4:**
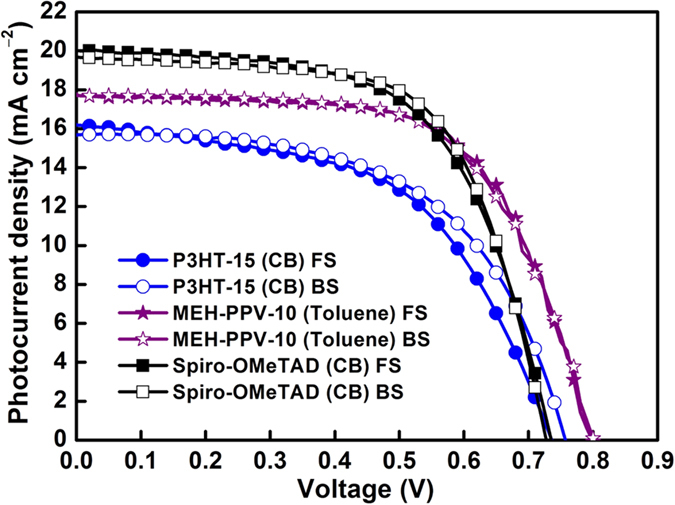
Current density–voltage (*J–V*) characteristic curves of the perovskite solar cells with Spiro-OMeTAD, P3HT, and MEH-PPV HTMs at forward scan (FS) and backward scan (BS).

The effects of solvents and concentration on device performance are investigated. Figure 3 shows the typical current density–voltage characteristics (*J–V*) measured under 100 mW cm^−2^ AM 1.5 solar illumination for solar cells with HTMs of 8 wt% Spiro-OMeTAD in chlorobenzene (CB), 15 mg mL^−1^ P3HT in CB, and 5–15 mg mL^−1^ MEH-PPV in CB, dichlorobenzene (DCB), and toluene. Table 1 summarizes the values of the device parameters, such as short-circuit current density (*J*_*SC*_), *V*_*OC*_, fill factor (FF), and PCE (*η*) values, and both the one- and two-step methods for preparing the perovskite layer were tested. However, because the one-step method demonstrates more general applicability in this work, it is used for preparing the perovskite layer; it was systematically investigated to examine the effect of solvents on the use of Spiro-OMeTAD, P3HT, and MEH-PPV based on one-step method. We also prepared the devices based on the planar structure (without any mesoporous TiO_2_ layer) by the one-step method, as shown in Figure S2 in the Supplementary Information. Since the planar structure one always comes with a huge hysteresis, we decided to use the devices structure with a mesoporous TiO_2_ layer. The effect of the HTM thickness of MEH-PPV (Fig. 2f,g) was examined by controlling the variation of the CB concentration. PPV was insoluble in most solvents as its backbone comprises rigid monomer units; however, its derivative, hairy-rod polymer MEH-PPV, was highly soluble in common organic solvents, such as THF, chloroform, chlorobenzene, dichlorobenzene, toluene, and xylene as its backbone comprises short flexible soft side chains^33^. Nevertheless, at a MEH-PPV concentration of higher than 15 mg mL^−1^, the viscosity of the MEH-PPV solution became so high that it was difficult to spread its solution on perovskite so as to achieve a completely covered layer. In the MEH-PPV/toluene solution, toluene molecules are among the conjugated main chains, and they prevent segment aggregation from the inter-conjugated main chains. Previously, some studies have demonstrated that the charge carrier mobility in toluene-cast MEH-PPV films is higher than that of CB-cast MEH-PPV films^34^,^35^. The MEH-PPV/toluene-based device with an optimized thickness or concentration at 10 mg mL^−1^ exhibited the highest *η* of 8.87%. The low *V*_*OC*_ for Spiro-OMeTAD can be explained by the fact that Spiro-OMeTAD (−5.1 eV) has a HOMO higher than that of MEH-PPV (−5.4 eV) (Fig. 1). As for the hysteresis behavior of *J–V* performance, MEH-PPV-based cells exhibited good hysteresis-free *J–V* characteristics, while P3HT-based cells and Spiro-OMeTAD cells exhibited small hysteresis between forward and back scans (Fig. 4).

**Figure 5:**
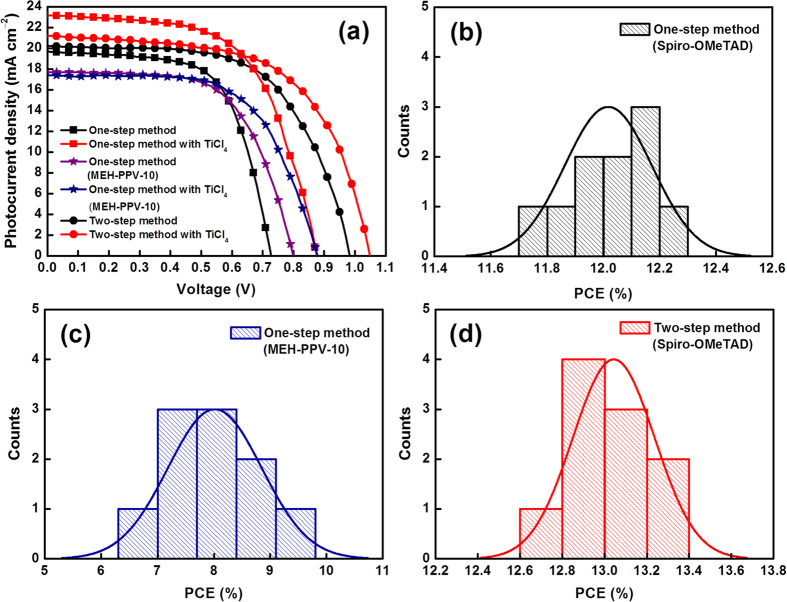
(**a**) Current density–voltage (*J–V*) curves of perovskite solar cells with Spiro-OMeTAD and MEH-PPV as the HTMs by one- and two-step methods for preparing perovskite with or without post-treatment using TiCl_4_. PCE histograms from the one-step method with TiCl_4_ post-treatment for (**b**) Spiro-OMeTAD, (**c**) MEH-PPV, and (**d**) PCE histograms from the two-step method with TiCl_4_ post-treatment for Spiro-OMeTAD with 10 cells.

For further enhancing device performance, the two-step method for preparing the perovskite layer was utilized, including post-treatment of the mesoporous TiO_2_ layer using TiCl_4_ before the deposition of the perovskite layer. In a dye-sensitized solar cell (DSSC) system, TiCl_4_ post-treatment improves the adhesion and mechanical strength of the nanocrystalline TiO_2_ layer and enhances the surface roughness factor and necking of the TiO_2_ particles, thereby increasing dye adsorption and resulting in high photocurrent^36^. Figure 5 and Table 2 show the *J–V* curves, histogram with the Gaussian fit of PCE distribution, and photovoltaic characteristics of perovskite solar cells fabricated using Spiro-OMeTAD and MEH-PPV as the HTMs by one- and two-step methods for preparing the perovskite layer with or without post-treatment using TiCl_4_. From the results, *J*_*SC*_ and *V*_*OC*_ significantly increased after post-treatment of the mesoporous TiO_2_ layer using TiCl_4_ for Spiro-OMeTAD; this increase in *V*_*OC*_ and *J*_*SC*_ is attributed to the blocking of recombination at the interface between perovskite and the FTO interface and post-treatment using TiCl_4_, respectively. The best performance for MEH-PPV-based devices was observed after fabrication by the one-step method for preparing the perovskite layer with post-treatment of the mesoporous TiO_2_ layer using TiCl_4_ with a *η* of 9.65%. The devices fabricated by the one-step method always exhibited high *J*_*SC*_, while devices fabricated by the two-step method always exhibited high *V*_*OC*_ compared to each other in both methods. This result is attributed to either the difference in the morphologies of perovskite observed between the one- and two-step methods or the incorporation of Cl residues in the perovskite crystals. The best performance for the Spiro-OMeTAD-based HTMs was observed by the two-step method for preparing the perovskite layer with post-treatment of the mesoporous TiO_2_ layer using TiCl_4_, achieving an *η* of 13.38%, a *J*_*SC*_ of 21.19 mA cm^−2^, a *V*_*OC*_ of 1.04 V, and an FF of 0.61 (backward scan), albeit with presence of small hysteresis.

**Figure 6:**
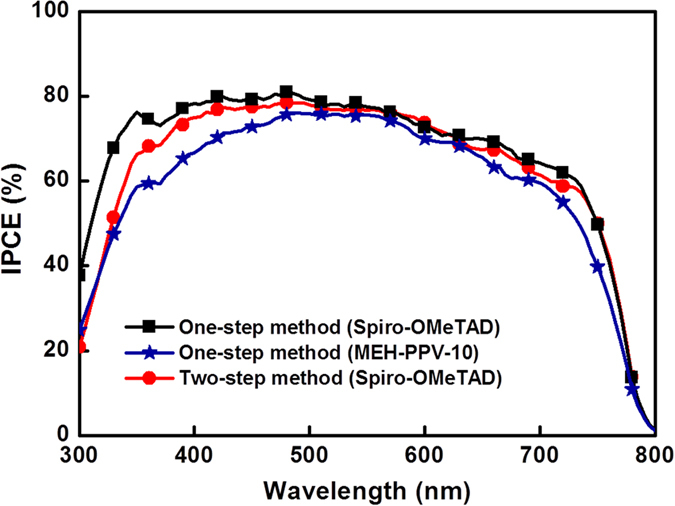
Incident-photon-to-current-efficiency (IPCE) spectra of perovskite solar cells with Spiro-OMeTAD and MEH-PPV as the HTMs by the one- or two-step method utilized for preparing perovskite layers.

Figure 6 shows the incident-photon-to-current-efficiency (IPCE) spectra of the perovskite solar cell. As can be observed, the generation of photocurrent began at 800 nm, which is in agreement with the bandgap of CH_3_NH_3_PbI_3_, and attained peak values of greater than 80% in the short-wavelength region of the visible spectra. These spectra indicate that the difference in *J*_*SC*_ between one- and two-step methods for the preparation of perovskites with Spiro-OMeTAD is in full light absorption wavelength of perovskite, and as compared to the Spiro-OMeTAD device, the MEH-PPV-based device exhibited a lower IPCE ranging from 300 to 500 nm, attributed to the light absorption of MEH-PPV (400–500 nm) in this region^29^. Although the cell performance of polymeric MEH-PPV was not better than that of Spiro-OMeTAD, their similar spectra still supports the view that MEH-PPV is an excellent HTM for perovskite solar cells. Notably, the conversion efficiency of the solar cells fabricated by the two-step method was greater than those by the one-step methods as reported previously, attributed to the submicrometer-sized CH_3_NH_3_PbI_3_ tetragonal crystals and the perfectly infiltrated PbI_2_ into the TiO_2_ mesoporous network^2^.

**Figure 7:**
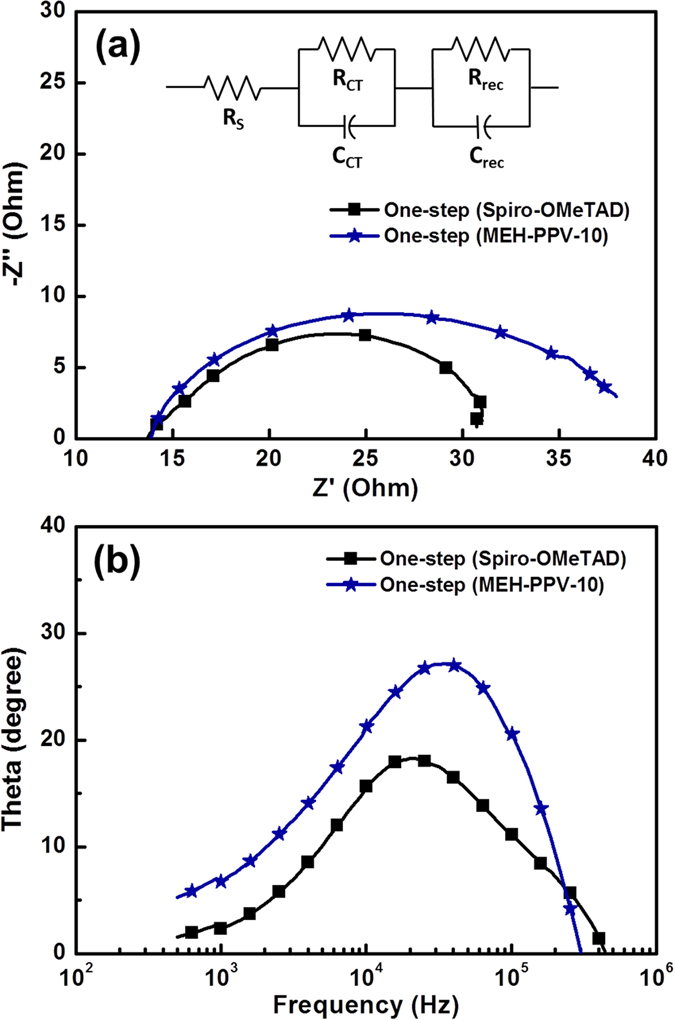
Electrochemical impedance spectra (EIS), (**a**) Nyquist plot and (**b**) Bode plot for the perovskite solar cells with Spiro-OMeTAD and MEH-PPV as HTM at one-step or two-step method for perovskite layer preparation with TiCl_4_ post-treatment on mesoporous TiO_2_ layer. The equivalent circuit of this study is shown in the inset.

Electrochemical impedance spectroscopy (EIS) was employed for quantifying the parameters of charge transport in solar cells, such as chemical capacitance, recombination resistance, and charge conductivity. These parameters are imperative to aid in the explanation of the factors that determine the performance metrics of the HTMs in the corresponding solar cells, and Table 2 summarizes the *V*_*OC*_, FF, and *J*_*SC*_ values. Figure 7 shows the (a) Nyquist and (b) Bode plots of the solar cells with different HTMs, which were recorded at *V*_*OC*_ under 1 sun illumination. The frequency in EIS measurements ranged from 1 Hz to 1 MHz. The inset of Fig. 7a shows the fitting of the EIS spectra to an appropriate equivalent circuit model. Typically, Nyquist plots (Fig. 7a) were separated into two main regions or arcs at high and low frequencies; nevertheless, only one arc was observed under illumination. For convenience, a parallel charge-transfer resistance (R_CT_)–chemical capacitance (C_CT_) sub-circuit was applied, where recombination resistance (R_rec_) and chemical capacitance (C_rec_) represent the charge recombination resistance and capacitance, respectively. Here, the absence of the recombination of electrons through a TiO_2_ network is attributed to the very thin TiO_2_ films and low electron transport resistance^15^. Hence, only one arc typically originates from the charge-transfer resistance at the interface between perovskite and HTMs in the devices. In accordance to a previous study on CH_3_NH_3_PbI_3_, a simplified circuit model (see inset of Fig. 7a) was utilized, and the arc was assigned to the charge-transfer resistance in the devices^15^. As the devices predominantly differ with respect to the HTM, the arc is attributed to R_CT_ and C_CT_ at the interface between perovskite and HTMs. After photogenerated carriers were separated, holes transfer in the HTMs, and electrons transfer in the TiO_2_ layer before arriving at both the electrodes. Hence, the charge-transfer resistance is one of the main factors limiting cell performance. From Table 2, as compared to the MEH-PPV based device, the Spiro-OMeTAD-based device exhibited a higher J_SC_ under 1 sun illumination, indicative of better contact between Spiro-OMeTAD and perovskite as compared with that between MEH-PPV and perovskite. Hence, the charge-transfer resistance R_CT_ simultaneously decreases because of better contact and easier charge transfer at the interface between perovskite and Spiro-OMeTAD. In addition, the electron lifetimes (τ_e_) for the Spiro-OMeTAD and MEH-PPV-based devices were determined by Bode-phase plots. Figure 7b shows the Bode-phase plots of EIS spectra, which show the frequency peaks corresponding to charge transfer at different interfaces of Spiro-OMeTAD and MEH-PPV-based devices. From Fig. 7b and Table 2, the characteristic frequency peaks exhibited a shift toward lower frequencies for Spiro-OMeTAD-based devices as compared to MEH-PPV-based devices, implying a longer electron lifetime is observed for Spiro-OMeTAD-based devices. These observations imply that a better contact and charge transfer are observed between Spiro-OMeTAD and the perovskite layer.

## Conclusion

Spiro-OMeTAD was systematically investigated in comparison to the well-known hole-transporting polymer material MEH-PPV for solution-processed perovskite solar cells. The best performance for MEH-PPV was achieved by the one-step method for preparing the perovskite layer by post-treatment of the mesoporous TiO_2_ layer using TiCl_4_, with an *η* of 9.65% as well as hysteresis-free performance. The best performance for Spiro-OMeTAD was observed by the two-step method prepared using TiCl_4_-treated perovskite, exhibiting an *η* up to 13.38%. Our study demonstrated that MEH-PPV as a cost-effective, dopant-free HTM is a potential candidate for the development of perovskite solar cells in the future. Furthermore, as compared to Spiro-OMeTAD-based devices, which were typically affected by moisture, MEH-PPV-based devices without encapsulation exhibited higher stability against ambient moisture, and we will further disclose it in other study.

## Methods

### Synthesis of methylammonium iodide

Methylammonium iodide (CH_3_NH_3_I) was prepared from 24 mL of methylamine solution (33% in ethanol) and diluted with 100 mL of absolute ethanol. A 10 mL aqueous solution of hydriodic acid (57 wt%) was added to this solution under constant stirring. After a reaction time of 1 h at room temperature, the solvents were removed by rotary evaporation. The obtained white solid was washed with dry diethyl ether and finally recrystallized from ethanol.

### Device fabrication

FTO coated glass sheets (10 Ω □^−1^, Nippon Sheet Glass, 1.1 mm in thickness) were etched with zinc powder and HCl (2.0 M) to obtain the required electrode pattern. The sheets were then washed with 100 mL soap (2% Hellmanex^®^ II in water), deionized water, acetone, and 2-propanol and finally treated under UV ozone for 10 min to remove the last traces of organic residues. To make a dense TiO_2_ compact layer, the cleaned FTO glasses were coated with 0.15 M titanium diisopropoxide bis(acetylacetonate) (75% Aldrich) in 2-propanol (Wako) solution by the spin-coating method, and heated at 125 °C for 5 min. After the coated film was cooled down to the room temperature, the same process was repeated twice with 0.3 M titanium diisopropoxide bis(acetylacetonate) solution in 2-propanol. The three times coated FTO glasses with TiO_2_ precursor solutions were heated at 550 °C for 15 min. The substrates were then coated with a commercially available screen-printable TiO_2_ paste (Solaronix, T/SP) mixed in 2:7 weight ratio with ethanol, prepared and sintered at 550 °C in air to achieve mesoporous TiO_2_ films. Afterward, the sintered TiO_2_ films were immersed in 0.02 M aqueous TiCl_4_ (Aldrich) solution at 70 °C for 60 min, and heated at 550 °C for 30 min. For one-step method, the CH_3_NH_3_PbI_3_ perovskite precursor solution (CH_3_NH_3_I to PbI_2_ in 3:1 molar ratio, Sigma Aldrich 98%) was prepared to a concentration of 40 wt% in N-dimethylformamide (DMF), and spin coated onto the substrate under ambient atmosphere. The substrate was then transferred to a hotplate where it was heated in inert atmosphere to 100 °C for 90 min. This procedure allows for the formation of a perovskite layer in the mesoporous TiO_2_ film. For two-step method, the mesoporous TiO_2_ film were infiltrated with PbI_2_ by spin-coating a PbI_2_ solution in DMF (462 mg ml^−1^) that was kept at 70 °C for 30 min. After drying, the films were dipped into a solution of CH_3_NH_3_I in 2-propanol (10 mg ml^−1^) for 45 s and rinsed with 2-propanol for 10 s. After drying, the films were covered with a 400 nm layer of Spiro-OMeTAD (Merck). 96 mg of Spiro-OMeTAD was dissolved in 1 mL of chlorobenzene (CB) and mixed with 10 μL 4-tertbutylpyridine (tBP) and 40 μL of a 170 mg mL^−1^ bis(trifluoromethane)sulfonimide lithium salt (LiTFSI) solution in acetonitrile (ACN). This solution was spin coated at 3000 rpm for 45 s. In the second case, the hole-transport layer was obtained by a spin-coating in nitrogen atmosphere (glove box), poly(3-hexylthiophene-2,5-diyl) (P3HT, Rieke Metals, Inc., 15 mg mL^−1^, MW 50,000~70,000) solution in CB and 5-(2′-ethyl-hexyloxy)-p-phenylenevinylene (MEH-PPV, American Dye Source, Inc., MW 100,000~150,000, 5~15 mg mL^−1^) solution in CB, dichlorobenzene (DCB), or toluene with the following parameters: 600 rpm for 12 s and finally at 1500 rpm for 40 s; dried up at 90 °C for 10 min and 120 °C for 10 min. Before thermal depositing the gold electrodes, Spiro-OMeTAD was treated for oxidative doping in air overnight at room temperature and 30% relative humidity to enhance the conductivity; however, films of P3HT and MEH-PPV were employed as dopant-free without oxidation.

### Solar cell characterization

Photocurrent density–voltage (*J–V*) characteristics were measured by a computer-controlled digital source meter (Keithley 2400) under 1 sun illumination. Irradiation of 100 mW cm^−2^ (1 sun) light was made with a Peccell Technologies, Inc. PEC-L11 solar simulator (AM 1.5G). All photovoltaic devices were masked with a thin black mask which was used to define the active area to be 0.1032 cm^2^. Bias voltage scanning was carried out at step voltage: 0.01 V; number of power line cycle (NPLC): 1 (20 ms); search delay: 0.05 s at scanning rate of 140 mV s^−1^. The incident photon to current efficiency (IPCE), or external quantum efficiency (EQE) action spectra of the device were measured by Keithley 2400 source meter in ambient air at room temperature on Peccell Technologies, Inc., PEC-S20 by scanning wavelength with 5 nm interval in conditions of a delay time of 5000 ms and under short-circuit condition (0 bias) of device. Absolute incident power density of monochromatic light in EQE measurement was monitored based on a standard Si photodetector.

## Additional Information

**How to cite this article**: Chen, H.-W. *et al*. Efficiency Enhancement of Hybrid Perovskite Solar Cells with MEH-PPV Hole-Transporting Layers. *Sci. Rep.* **6**, 34319; doi: 10.1038/srep34319 (2016).

## Supplementary Material

Supplementary Information

## Figures and Tables

**Table 1:** Photovoltaic characteristics, *J*_*SC*_, *V*_*OC*_, FF, and *η* of perovskite solar cells with Spiro-OMeTAD, P3HT, and MEH-PPV as HTMs dissolved in various solvents as well as concentrations.

HTM concentration (mg mL^−1^)	*J*_*SC*_ (mA cm^−2^)	*V*_*OC*_ (V)	FF	*η* (%)
Spiro-OMeTAD (CB)	19.68	0.73	0.64	9.20
P3HT-15 (CB)	15.68	0.76	0.57	6.74
MEH-PPV-5 (CB)	13.58	0.79	0.64	6.88
MEH-PPV-10 (CB)	15.62	0.80	0.64	7.21
MEH-PPV-15 (CB)	2.96	0.76	0.22	0.49
MEH-PPV-10 (DCB)	16.18	0.80	0.62	8.06
MEH-PPV-10 (Toluene)	17.70	0.80	0.63	8.87

CB: chlorobenzene, DCB: dichlorobenzene, one-step method for preparation of perovskite.

**Table 2:** Photovoltaic characteristics, *J*_*SC*_, *V*_*OC*_, FF, and *η* of perovskite solar cells with Spiro-OMeTAD and MEH-PPV as the hole-transporting layer (HTL) by the one- and two-step methods for preparing perovskite with or without post-treatment by TiCl_4_ measured under 1 sun illumination.

	*J*_*SC*_(mA cm^−2^)	*V*_*OC*_ (V)	FF	*η* (%)	R_CT_ (Ω)	τ_e_ = 2πf^−1^ (s)
One-step method	19.68	0.73	0.64	9.20	—	—
One-step method with TiCl_4_	23.18	0.87	0.61	12.26	19.97	7.91 × 10^−6^
One-step method (MEH-PPV)	17.70	0.80	0.63	8.87	—	—
One-step method with TiCl_4_ (MEH-PPV)	17.36	0.88	0.63	9.65	24.53	4.98 × 10^−6^
Two-step method	20.24	0.98	0.62	12.23	—	—
Two-step method with TiCl_4_	21.19	1.04	0.61	13.38	21.45	—
