# Supplementary material for: Efficiency Enhancement of Hybrid Perovskite Solar Cells with MEH-PPV Hole-Transporting Layers
Source: Sci Rep. 2016 Oct 4;6:34319. doi: 10.1038/srep34319 (PMC5048417; doi:10.1038/srep34319)
Supplement: Supplementary Information [file srep34319-s1.pdf]

Supplementary Information

Efficiency Enhancement of Hybrid Perovskite Solar Cells with MEH-PPV Hole-Transporting Layers

Hsin-Wei Chen[1], Tzu-Yen Huang[1,2], Ting-Hsiang Chang[1], Yoshitaka Sanehira[3], Chung-Wei Kung[1], Chih-Wei Chu[2], Masashi Ikegami[3], Tsutomu Miyasaka[3,], and Kuo-Chuan Ho[1,*]**

[1]Department of Chemical Engineering, National Taiwan University, Taipei 10617, Taiwan

[2]Research Center for Applied Sciences, Academia Sinica, Taipei 11529, Taiwan

[3]Graduate School of Engineering, Toin University of Yokohama, Yokohama 1614, Japan

[*]corresponding author: kcho@ntu.edu.tw; Tel: +886-2-2366-0739

[**]corresponding author: miyasaka@toin.ac.jp; Tel: +81-45-974-5055

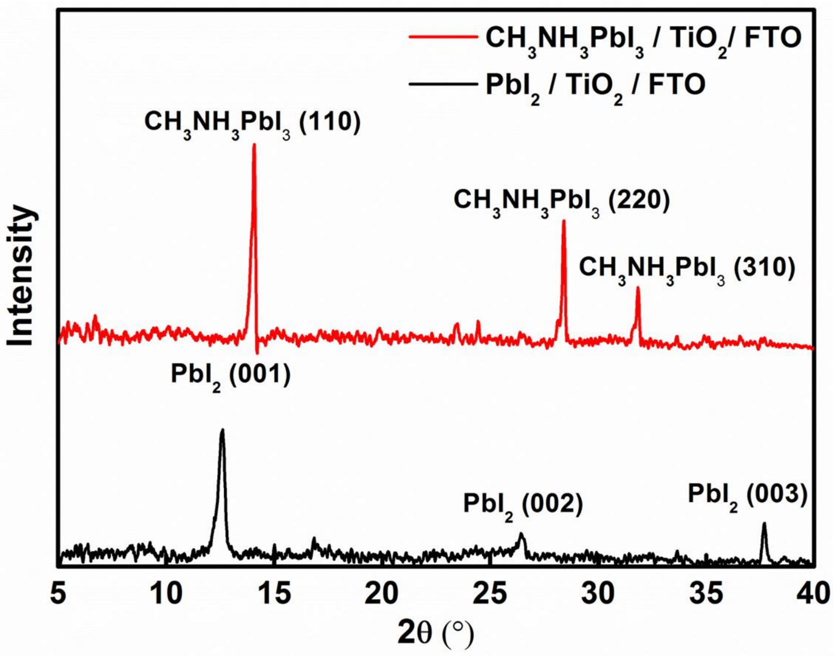

Supplementary Figure S1. XRD patterns of PbI_2/TiO_2 film and $CH_3NH_3PbI_3/TiO_2$ film after dipping of PbI_2/TiO_2 into a CH_3NH_3I containing solution.

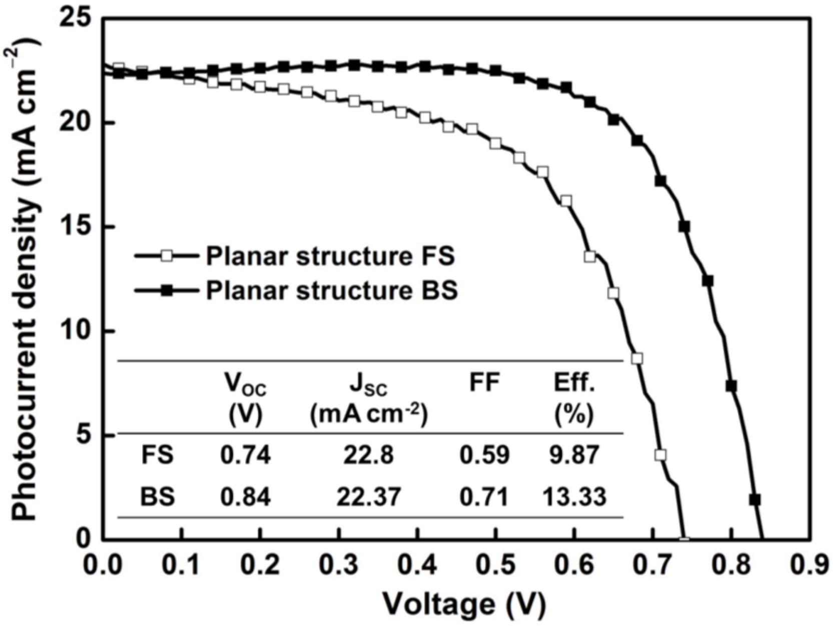

	V_{OC} (V)	J_{SC} (mA cm^{-2})	FF	Eff. (%)
FS	0.74	22.8	0.59	9.87
BS	0.84	22.37	0.71	13.33

Supplementary Figure S2. Current density–voltage (*J*–*V*) characteristic curves of perovskite solar cells in planar structure with Spiro-OMeTAD as a hole-transporting layer.
